# Raman spectroscopic study of ZnO/NiO nanocomposites based on spatial correlation model†

**DOI:** 10.1039/c9ra04555d

**Published:** 2019-08-28

**Authors:** Sarang Dev G., Vikas Sharma, Ashish Singh, Vidushi Singh Baghel, Masatoshi Yanagida, Atsuko Nagataki, Neeti Tripathi

**Affiliations:** Department of Physics, School of Physical Sciences (SoPS), Doon University Dehradun – 248001 Uttarakhand India vsharma.ph@doonuniversity.ac.in neetidtripathi@gmail.com; School of Materials Science and Technology, Indian Institute of Technology (IIT) BHU Varanasi – 221005 India; Global Research Center for Environment and Energy based on Nanomaterials Science (GREEN), National Institute of Materials Science (NIMS) Tsukuba Ibaraki 305-0044 Japan

## Abstract

The effect of nickel concentration has been investigated in ZnO/NiO nanocomposites synthesized using the co-precipitation method. The X-ray diffraction and TEM measurements confirm the distinct phase of NiO in the ZnO/NiO samples. Furthermore, the Raman study shows the sharp modes at 99 cm^−1^ and 438 cm^−1^ corresponding to E^(low)^_2_, E^(high)^_2_ of hexagonal wurtzite ZnO structure and, 1080 cm^−1^ associated to the two-phonon (2P) mode of NiO, respectively. We also compared the effect of Ni concentration on the formation of ZnO/NiO by analyzing E^high^_2_ Raman mode of ZnO with the help of spatial correlation model. The correlation lengths, broadening and asymmetry ratio obtained from the fitting showed good agreement with the experimental results.

## Abstract 2

Raman scattering of pure ZnO and ZnO/NiO nanocomposite have been investigated. Raman line shapes of the E^(high)^_2_ mode have been analyzed using the spatial correlation model.

## Graphical abstract

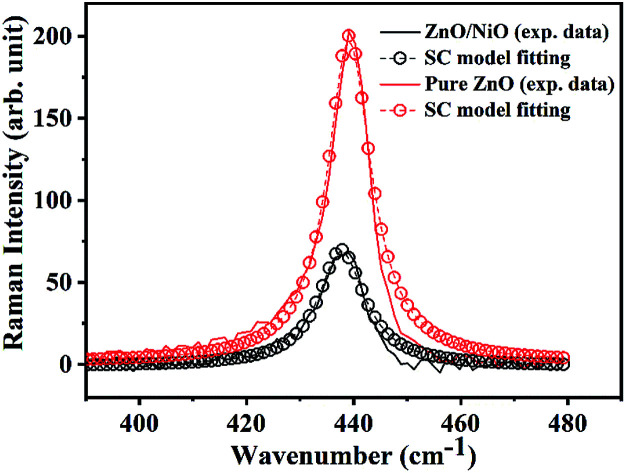

## Introduction

Over the years, zinc oxide (ZnO) has attracted tremendous interest in the field of photovoltaics, photocatalysis, supercapacitor and spintronics applications.^1,2^ It is now well established that the structural, optical, and electrical properties can be modified by intentional doping of the metal ions into the ZnO host matrix. While extensive studies have been devoted to the partial substitution of Zn^2+^ with the transition metal ions into the ZnO host matrix,^2^ the nanocomposite materials showed better chemical and mechanical stability, higher electrical conductivity and new interface properties in comparison with the separate phases.^3^ Therefore nanocomposites with unique properties physical and chemical properties can be applied in diverse fields, including medicine, energy, and ecology. Particularly, ZnO–NiO nanocomposites showed interesting properties due to the fact that n-type ZnO and p-type NiO build-up an inner electric field at the p–n junction interface, which possesses superior functional performances in comparison to the properties of individual phases. For example, Li *et al.*,^4^ observed much higher photocatalytic activity of ZnO/NiO heterojunction nanofibers than that of pure ZnO and NiO nanofibers due to highly efficient separation of photogenerated electron–hole pairs. Similarly, Zhou *et al.*,^5^ demonstrated a higher value of short-circuit current density (*J*_SC_) of 22.34 mA cm^−2^ and a PCE of 7.01%, compared to that of isolated ZnO/NiO photoanodes. Recent study showed, that the ZnO/NiO nanocomposites exhibit resistive switching properties which are exploited in the fabrication of Resistive Random-Access Memories (ReRAMs).^6^ Though, significant attention has been paid to the morphological evolution of the ZnO/NiO composites,^7–9^ a detail understanding about the structural disorder and defects is particularly of interest from both, theoretical and experimental point of view. Since Raman scattering study is based on the inelastic scattering due to lattice phonons, the nature of the material structure and structural disorder and, can be investigated using the Raman spectroscopic techniques.

In this paper, we investigated the structural evolution of ZnO/NiO nanocomposite at varying Ni concentrations. Samples were synthesized *via* co-precipitation method^10^ with varying Ni concentrations *i.e.*, 0.10 M, 0.20 M and 0.30 M. Samples were characterized using X-ray diffraction (XRD), transmission electron microscopy (TEM) and Raman spectroscopy. We further employed the spatial correlation (SC) model^11^ on the experimentally observed Raman scattering data, to attain the important information regarding the nature of the solid on a scale of the order of a few lattice constants. Therefore, an insight towards the material properties in terms of correlation lengths and asymmetry constants has been presented.

## Experimental details

Zinc acetate [(CH_3_COO)_2_Zn·2H_2_O] (Fisher Scientific) and nickel acetate tetrahydrate [C_4_H_6_NiO_4_·4H_2_O] (SRL Chemicals) were used as the starting materials. The precursor solutions were prepared by dissolving the zinc acetate and nickel acetate in 100 ml deionized water. Solutions were then placed on a magnetic stirrer and the temperature was maintained at 80 °C. The pH of the solution was maintained around 10, by adding the NaOH solution dropwise to the precursor solution. The stirring was continued for further 1 hour and then the samples were allowed to cool down. The cooled samples were washed with deionized water several times to remove the free ions and then filtered out by the filtration apparatus. The filtered samples were then dried in the hot air oven at 80 °C for 4 hours and annealed at 450 °C for 2 hours in muffle furnace under air ambience. Similar procedure was repeated for three nickel acetate molar concentrations *i.e.*; 0.10 M, 0.20 M and 0.30 M.

The structure of the ZnO/NiO nanocomposites was investigated using an X-ray diffractometer [Bruker D8 Advanced] with Cu Kα radiation (*λ* = 1.534 Å). A detailed information about the crystalline quality were gathered by using a high resolution Transmission Electron Microscope (TEM) equipped with a FEG source using FEI Tecnai TF20 TEM operated at 200 keV. The Raman spectra of the samples were recorded (model: Raman Touch-VIS-NIR, Nanophoton) at room temperature in *XY*-mapping mode using 488 nm laser wavelength at excitation power 1.02 mW and exposure time 10 000 ms. During the measurement the laser current was maintained 100% and the signals were recorded by a TE cooled back-thinned CCD camera.

Hereafter, the samples will be designated as ZnO, ZnO-1, ZnO-2 and ZnO-3 corresponding to the pure ZnO, Zn_0.9_Ni_0.1_O, Zn_0.8_Ni_0.2_O, and Zn_0.7_Ni_0.3_O respectively as shown in the Table 1.

**Table 1:** Sample description and parameters calculated from XRD

Sample code	Description	Crystallite size (nm)	Dislocation density	Strain (×10^−3^)
ZnO	ZnO	28.15	0.0355	7.885
ZnO-1	Zn_0.9_Ni_0.1_O	39.39	0.0253	5.636
ZnO-2	Zn_0.8_Ni_0.2_O	38.73	0.0258	5.732
ZnO-3	Zn_0.7_Ni_0.3_O	34.24	0.0292	6.482

## Results and discussion

**Fig. 1:**
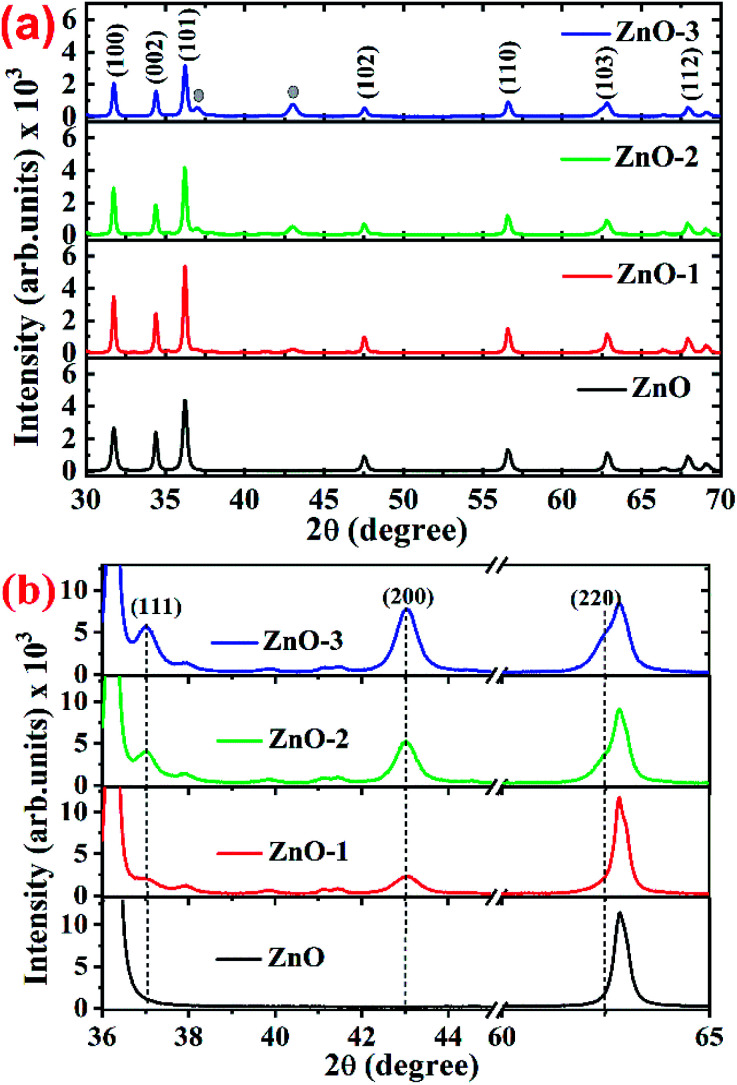
(a) Full 2*θ* range of XRD patterns for ZnO and ZnO/NiO samples, (b) extended 2*θ* range of XRD patterns for highlighting the NiO peaks in ZnO and ZnO/NiO samples.

**Fig. 2:**
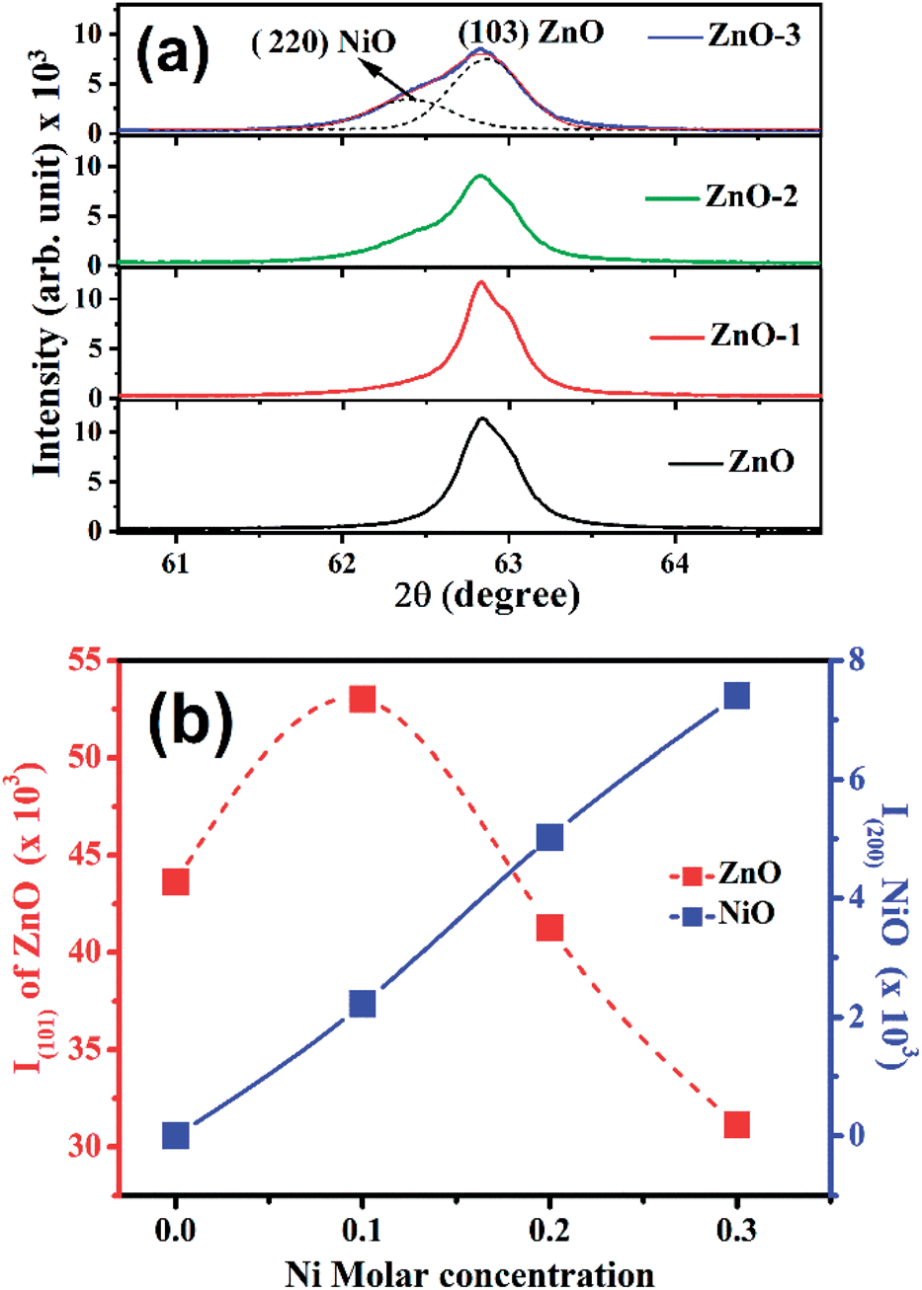
(a) Deconvolution of X-ray diffraction peak at 62.87° into two peaks *i.e.*, (220) NiO and (103) ZnO, (b) intensity dependence of ZnO (101) and NiO (200) peaks on the Ni molar concentrations.

Fig. 1(a) shows the XRD patterns of the pure ZnO and ZnO/NiO samples. The sharp peaks in the patterns indicate the highly crystalline nature of the samples. The diffraction peaks at various 2*θ* positions, can be indexed to (100), (002), (101), (102), (110), (103) and (112) hexagonal wurtzite structure of ZnO crystal.^3^ In all the samples other than pure ZnO, the traces of NiO were observed at 2*θ* positions 37.03 and 43.02 corresponding to the (111) and (200) planes of NiO fcc-cubic structure.^12^Fig. 1(b) showed the enlarged graph for the NiO peaks emerged after the addition of NiO in to the ZnO matrix. The de-convolution of XRD peak at 2*θ* position 62.87, shows the two contributions, one from ZnO (103) and another from NiO (220) plane for the samples ZnO-2 and ZnO-3, respectively, as shown in the Fig. 2(a). It is also observed that the intensity of NiO peaks increases linearly with increasing nickel concentration (Fig. 2(b)). The distinct features of NiO phase formation in all three Ni-doped ZnO samples indicated the ZnO/NiO nanocomposite formation. Further, the crystallite size (*D*) has been calculated using Debye–Scherrer formula (*D* = *kλ*/*β* cos *θ*),^10^ where *λ* is the wavelength of X-rays (1.534 Å), *θ* is the diffraction angle, *D* is the crystallite size, *k* (=0.9) is the shape factor and *β* is the full width at half maximum (FWHM) of the peak. The calculated crystallite size for each sample is as shown in the Table 1. There is a significant increase in the crystallite size, when the NiO is introduced into the ZnO sample. This can be due to the distortion produced because of the mismatch between the ionic radii of Zn^2+^ and Ni^2+^. But on further increase in the Ni concentration beyond ZnO-1, the nucleation is suppressed and the crystallite size is reduced subsequently. The strain in the samples is obtained by the equation *ε* = *β*/2 tan *θ*, where *β* is the FWHM of the peak and *θ* is the Bragg angle.^10,13^ The strain in the pure ZnO sample is calculated (given in the Table 1) to be 7.88 × 10^−3^ and reduced to a minimum of 5.63 × 10^−3^ for ZnO-1. On further increasing the Ni concentration, the strain increased up to 6.48 × 10^−3^ for ZnO-3. It is evident from, that the increased strain in the ZnO lattice due to Ni doping, causes the reduction in particle size. However, the strain of ZnO-1 is decreased from that of pure ZnO which may be due to the fact that the incorporated impurity has been trapped in non-equilibrium positions. As the concentration of Ni increases, the Ni^2+^ and the strain increases. The value of dislocation density (*δ*) is calculated using the relation *δ* = 1/*D*^2^,^10^ where *D* is the crystallite size (“*D*” values taken from the Table 1). It is observed that the dislocation density is decreased to a minimum for the sample ZnO-1 and then increased with the increase in the Ni concentration which is obvious from the strain calculations.

**Fig. 3:**
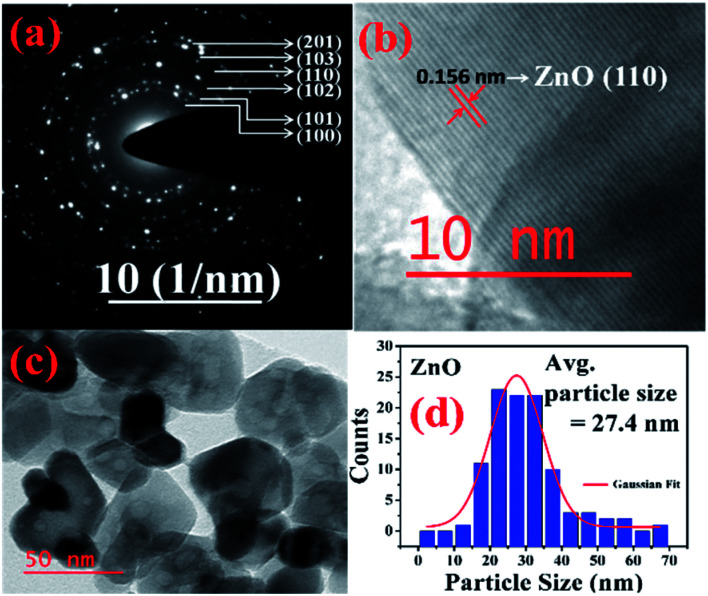
(a) SAED pattern, (b) HR-TEM, *d*-spacing (c) particle size (d) particle size distribution, of pure ZnO sample.

**Fig. 4:**
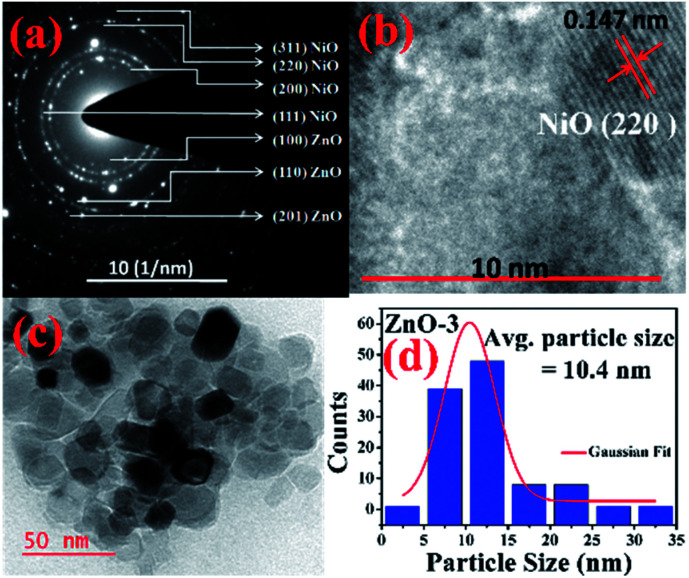
(a) SAED pattern, (b) HR-TEM, *d*-spacing (c) particle size (d) particle size distribution, of ZnO-3 sample.

More detailed information about the structure of the samples was further obtained from the TEM analysis. As seen in the Fig. 3(a), the selected area electron diffraction (SAED) patterns of the ZnO sample verifies polycrystalline feature of ZnO nanoparticles. The SAED ring patterns representing the planes (100), (101), (102), (110), (103), and (201) correspond to the hexagonal wurtzite ZnO structure and confirm the high crystallinity of the sample. In addition, the high resolution TEM image (HRTEM) shown in the Fig. 3(b) detects the lattice fringes of spacing 0.156 nm which corresponds to the (110) plane of wurtzite ZnO. It is also noticed from the Fig. 3(c) and other low magnification TEM images that the nanoparticles were formed mostly with hexagonal shape. Further, the size distribution of the particles was analysed with the help of a histogram of 100 particles given in the Fig. 3(d). The particle sizes determined from the TEM images range from 11 nm to 66 nm and from the Gaussian fit, the estimated average particle size is 27.4 nm for pure ZnO sample. For the sample ZnO-3 containing 0.30 M Ni, the SAED pattern is shown in the Fig. 4(a). Interestingly, along with the ZnO planes the diffraction rings corresponding to the (111), (200), (220), and (311) planes of the fcc-cubic phase of NiO were also detected here. This observation confirms the presence of separate NiO phase and the evidence of the ZnO/NiO nanocomposite formation. Fig. 4(b) shows a high resolution TEM micrograph depicting the fringes of spacing 0.147 nm corresponding to the (220) NiO plane. In comparison with the pure ZnO sample, the particle size distribution is narrow, in the case of ZnO-3 and the greater number of particles seem to possess similar shapes as shown in the low magnification micrograph in the Fig. 4(c). The histogram provided in the Fig. 4(d) explains the size distribution of particles ranging from 4 nm to 27 nm. The average particle size of the ZnO-3 sample is found to be around 10.4 nm using a Gaussian fit on the particle size histogram. The sizes calculated from TEM micrographs are quite smaller than the crystallite sizes from XRD. This can be understood as the breaking of agglomerates during the ultrasonic treatment while preparing the samples for TEM measurement on carbon coated grids. Nevertheless, SAED patterns and fringe patterns revealed the high quality ZnO/NiO nanocomposite samples, obtained *via* co-precipitation method.

**Fig. 5:**
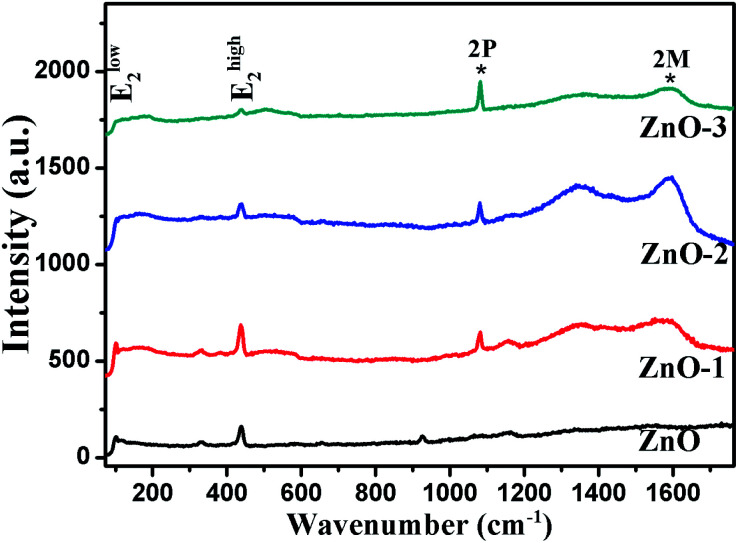
Raman spectra of ZnO and ZnO/NiO samples.

**Fig. 6:**
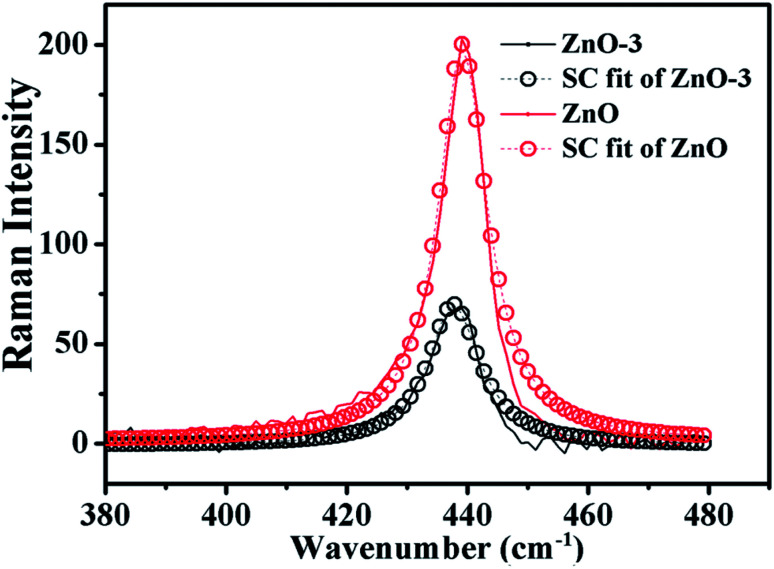
SC model fitting of the experimentally observed Raman data (E^high^_2_ Raman mode) for ZnO and ZnO-3 samples.

Raman spectroscopy was performed to confirm the crystallinity and structure of the samples. ZnO has a hexagonal wurtzite structure with one Zn ion surrounded tetrahedrally by four oxygen ions and *vice versa*.^14^ With four atoms per unit cell, it has three acoustic and nine optic phonon branches. The zone-center optical phonons can be classified according to the following irreducible representation: *Γ*_opt_ = A_1_ + E_1_ + 2E_2_ + 2B_1_, where B_1_ modes are silent, A_1_ and E_1_ are polar modes, both Raman and IR active, while E_2_ modes (E^low^_2_ and E^high^_2_) are nonpolar and Raman active only.^15,16^Fig. 5 shows the Raman spectra of all the four samples. The Raman modes at 99 cm^−1^ and 438 cm^−1^ corresponding to E^low^_2_ and E^high^_2_ respectively are representatives of wurtzite structure of good crystalline quality.^16,17^ The modes present at 1080 cm^−1^ and 1593 cm^−1^ show the two-phonon (2P) and two-magnon (2M) modes respectively corresponding to the NiO phase.^18^ Hereafter, the Raman study is concentrated on the analysis of E^high^_2_ mode, which is a characteristic of wurtzite ZnO crystal. The effects of disorder and Alloy Potential Fluctuations (APF) in the ZnO/NiO can be investigated using the Spatial Correlation (SC) Model or Phonon Confinement Model (PCM).^15,16,19,20^ In an ideal crystal, the spatial correlation function is infinite because of the momentum conservation (*q* = 0) of Raman scattering. Then the Raman lineshape would be symmetric and purely Lorentzian. However, any structural disorder in the crystal may destroy the symmetry in the Raman lineshape and can break the momentum selection rule leading to a finite phonon correlation length (*L*). Using the SC model, we can calculate the correlation length, which is physically interpreted as average grain size of the localized region. According to this model, Raman intensity (*I*(*ω*)) at a frequency *ω* can be written as:^16,19,20^
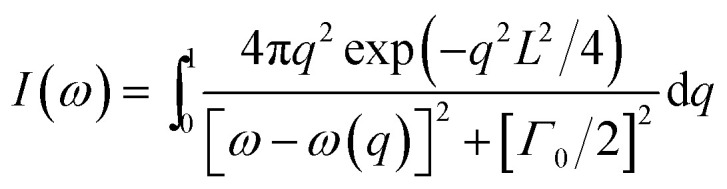
where *q* is the wave vector, expressed in the units of 2π/*a* (*a* is the lattice constant), *L* is the correlation length, *ω*(*q*) is the Raman phonon dispersion relation, and *Γ*_0_ is the intrinsic linewidth of the E^high^_2_ phonon mode. The dispersion relation for E^high^_2_ mode of the ZnO wurtzite structure is given by, *ω*(*q*) = *A* + *B* cos(π*q*), where *A* (=425.5 cm^−1^) is zone-center phonon frequency and *B* (=12.5 cm^−1^) is the difference between the zone-center and the zone-boundary frequencies of the phonon dispersion curve.^16,19^ Using the correlation length *L* as an adjustable parameter, we can get the value of *L* by fitting the Raman line-shape of E^high^_2_ mode. Fig. 6 shows the SC model fitted of E^high^_2_ mode for ZnO and ZnO-3 samples. The value of *L* is calculated as a fitting parameter in the SC model for all the samples, which is found to be in good agreement with the crystallite size observed from the XRD data as shown in the Table 2. The value of *L* increased with the molar concentration of Ni. This indicated that the phonon extended region became large in ZnO/NiO composite.

**Table 2:** The asymmetry ratio (*Γ*_a_/*Γ*_b_), FWHM (*Γ*) of E^high^_2_ mode and relative Raman shift (Δ*ω*) of the samples

Sample	Correlation length (*L*)	*Γ*_a_/*Γ*_b_	*Γ*	Δ*ω*
ZnO	28.4	0.657	10.01	0.90
ZnO-1	39.4	0.888	10.20	0.20
ZnO-2	38.6	1.092	9.79	0.60
ZnO-3	34.5	1.293	9.88	0.46

**Fig. 7:**
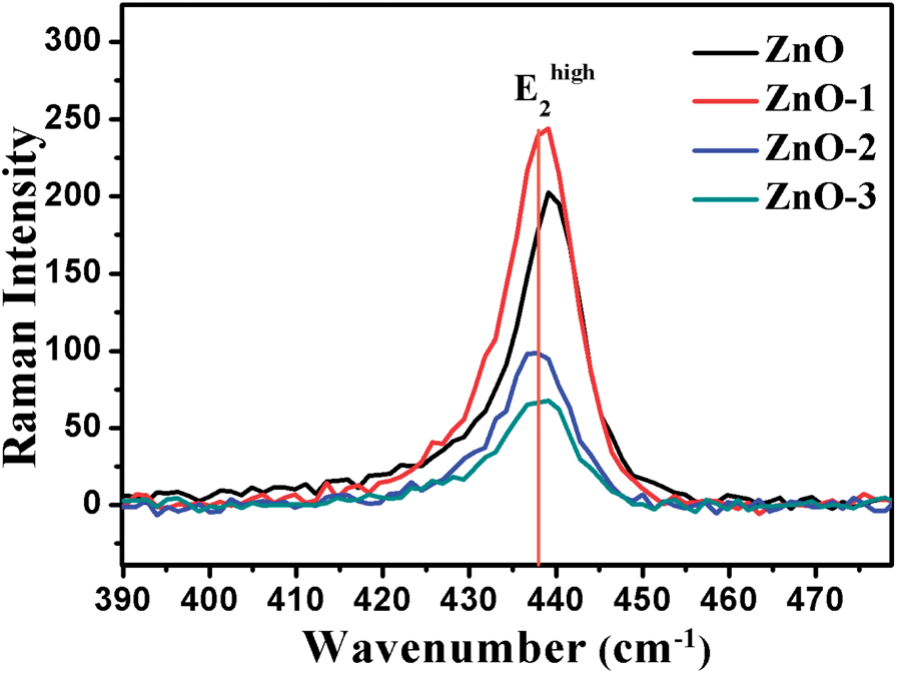
Raman shift in the E^high^_2_ mode for ZnO and ZnO/NiO samples.

**Fig. 8:**
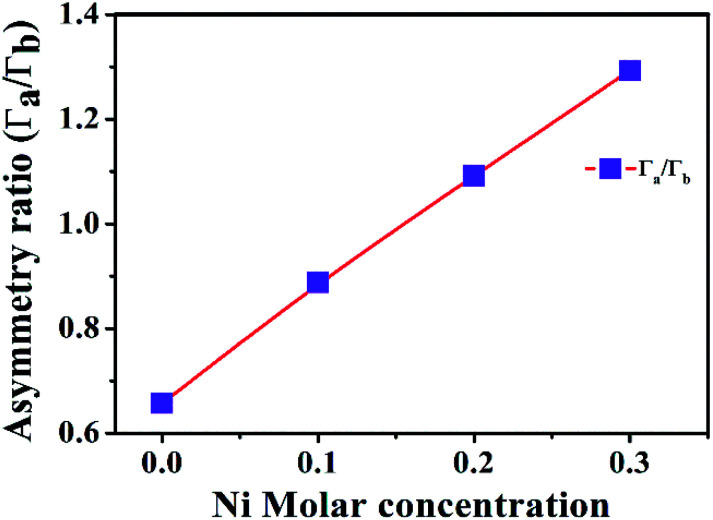
Variation of asymmetry ratio with Ni molar concentration.

Fig. 7 illustrates the Raman shift at the E^high^_2_ mode. A red shift in the E^high^_2_ Raman mode in ZnO/NiO samples indicates the tensile strain along the *c*-axis of the hexagonal wurtzite ZnO phase.^15^ Further, to analyse the disorder in more detail, we have calculated the broadening (*Γ*), the broadening asymmetry (*Γ*_a_/*Γ*_b_), and the shift (Δ*ω*) in the peak position of the Raman line shape at E^high^_2_ mode. The calculated parameters are tabulated in the Table 2 and the Fig. 8 shows the change in asymmetry ratio (*Γ*_a_/*Γ*_b_) as a function of Ni concentration in the ZnO/NiO samples. It is evident that the sample ZnO-1 is having the lowest asymmetry in the lineshape.

## Conclusions

ZnO and ZnO/NiO nanoparticles were synthesized using co-precipitation method. Distinguishable NiO phase was observed in the XRD, TEM and Raman spectroscopy, which suggested the formation of ZnO/NiO nanocomposites. XRD revealed, that the defects are created due to the incorporation of Ni in the ZnO lattice. A detailed insight of E^high^_2_ Raman mode showed that 0.10 M Ni concentration is optimal to obtain the maximum phonon correlation lengths with minimum asymmetry. The increased value of correlation length and lower asymmetry at 0.10 M Ni concentration inferred that, the phonon extended region became large with small amount of Ni addition in to ZnO matrix.

## Conflicts of interest

There are no conflicts to declare.

## Supplementary Material

RA-009-C9RA04555D-s001
