# Supplementary material for: Raman spectroscopic study of ZnO/NiO nanocomposites based on spatial correlation model
Source: RSC Adv. 2019 Aug 28;9(46):26956–60. doi: 10.1039/c9ra04555d (PMC9070446; doi:10.1039/c9ra04555d)
Supplement: RA-009-C9RA04555D-s001 [file RA-009-C9RA04555D-s001.pdf]

Electronic Supplementary Material (ESI) for RSC Advances.
This journal is © The Royal Society of Chemistry 2019

# Supplementary information

## Raman spectroscopic study of ZnO/NiO nanocomposites based on Spatial Correlation Model

Sarang Dev G[a], Vikas Sharma[a*], Ashish Singh[b], Vidushi Singh Baghel[a], Masatoshi Yanagida[c], Atsuko Nagataki[c], Neeti Tripathi[a,#*]

[a]Department of Physics, School of Physical Sciences (SoPS), Doon University, Dehradun - 248001, Uttarakhand, India

[b]School of Materials Science and Technology, Indian Institute of Technology (IIT) BHU, Varanasi – 221005, India

[c]Global Research Center for Environment and Energy based on Nanomaterials Science (GREEN), National Institute of Materials Science (NIMS), Tsukuba, Ibaraki 305-0044, Japan

[#] *Current Affiliation:* National Institute of Advanced Industrial Science and Technology (AIST), 1-8-31 Midorigaoka, Ikeda, Osaka 563-8577 Japan

e-mail: vsharma.ph@doonuniversity.ac.in, neetidtripathi@gmail.com

**XPS study:**

XPS was performed for the detailed understanding of elemental information and their oxidation states before and after the introduction of NiO phase. The full range XPS spectra of ZnO and ZnO-3 samples are shown in the figure S1, revealing the presence of Zn, O, Ni and C. Figure S2 shows the extended view for the $Zn_{2p}$, $Ni_{2p}$ and $O_{1s}$ peaks for sample ZnO and ZnO-3. The spectral lines observed at binding energies of 1020.62 eV (Zn $2p_{3/2}$) and 1045.60 eV (Zn $2p_{1/2}$), correspond to the doublet of Zn as shown in figure S2(a). The intensity of both the $Zn_{2p}$ peaks is found to be decreased due to excess structural disorder in ZnO-3 sample after introducing the NiO phase.  Figure S2 (b) illustrate the doublets: the Ni-$2p_{3/2}$ at 853 eV and the Ni-$2p_{1/2}$ at 871 eV.  A very clear peaks of Ni-2p lines can be observed for ZnO-3 sample. The peak located around 530-535 eV can be assigned to the 1s state of oxygen. The O1s peak observed at the lower binding energy 530.3 eV can be assigned to $O^{2-}$ ions in both Zn – O and Ni – O bonds, while the O1s peak located at the higher binding energy 534.7 eV can be assigned to $O^{-}$ and $O^{2-}$ ions in oxygen-deficient regions in the sample matrix as illustrated in figures S2 (c) and (d).

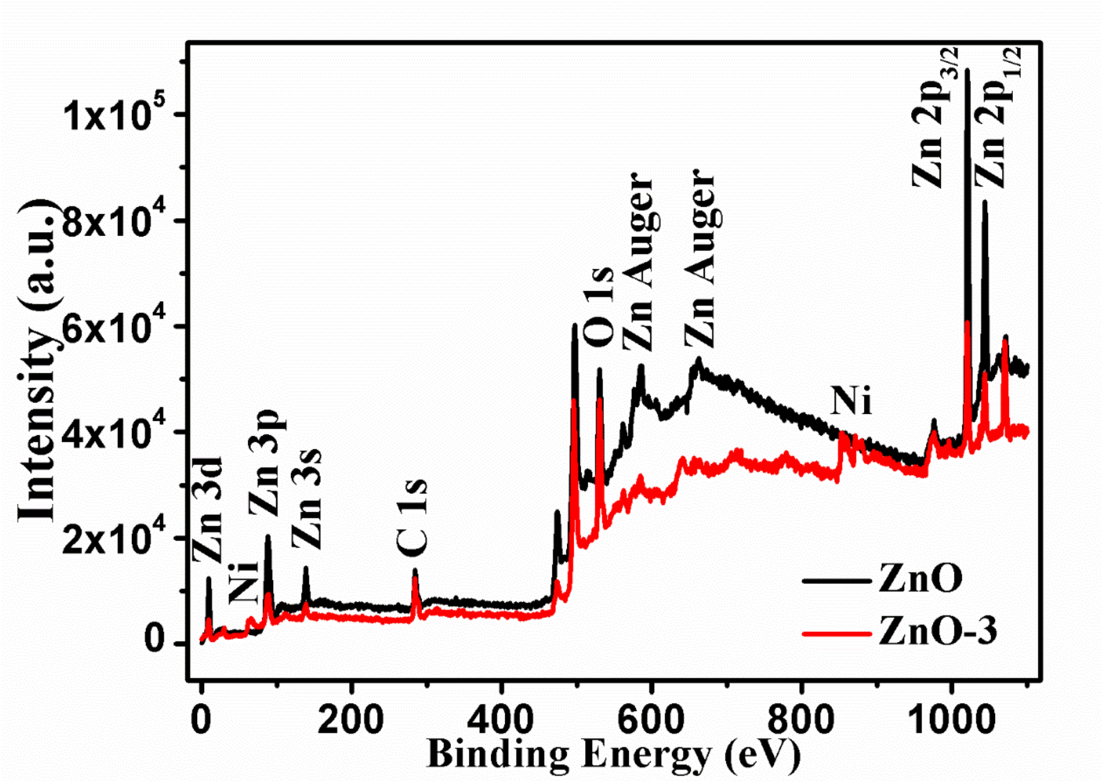


**Figure S1:** Full range XPS spectra of pure ZnO and ZnO-3 samples, showing the Zn, Ni and O1s peaks.

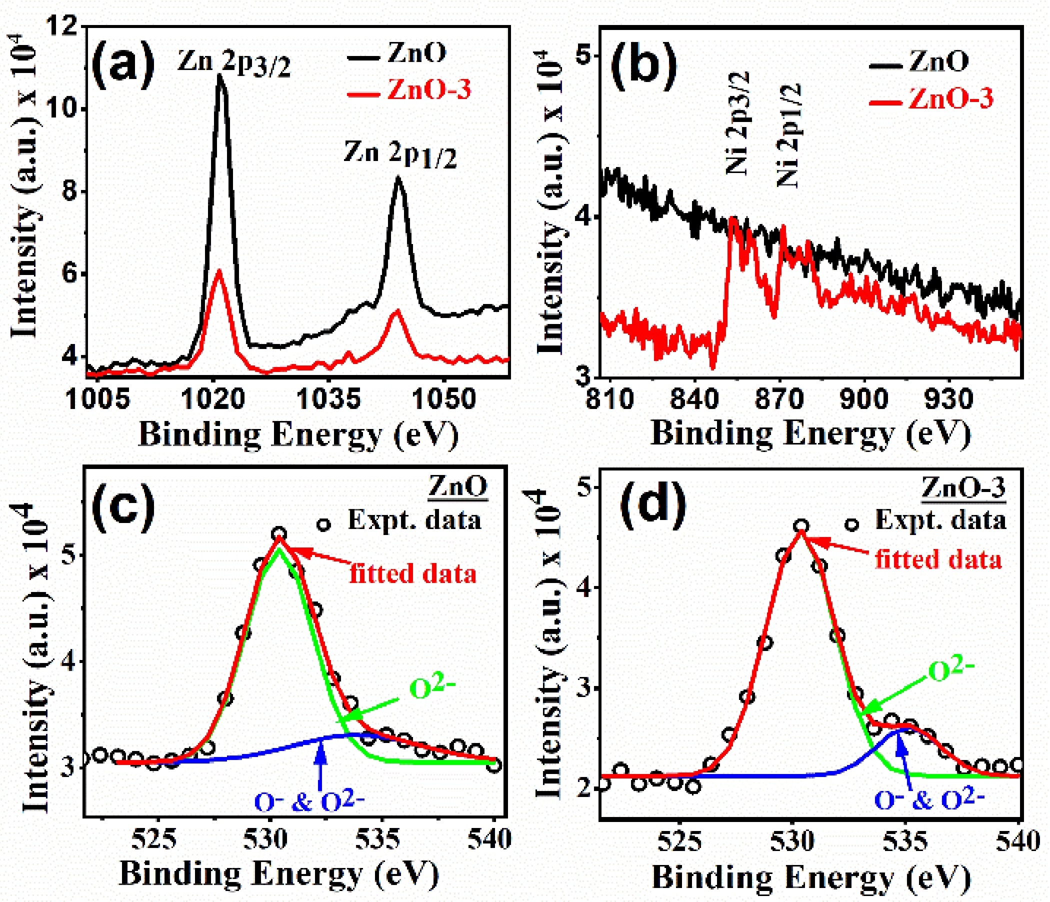


**Figure S2:** XPS spectra of (a): Zn 2p (b) Ni 2p (c ) O1s peaks for ZnO (d) O1s peak for ZnO-3.

## UV-visible study:

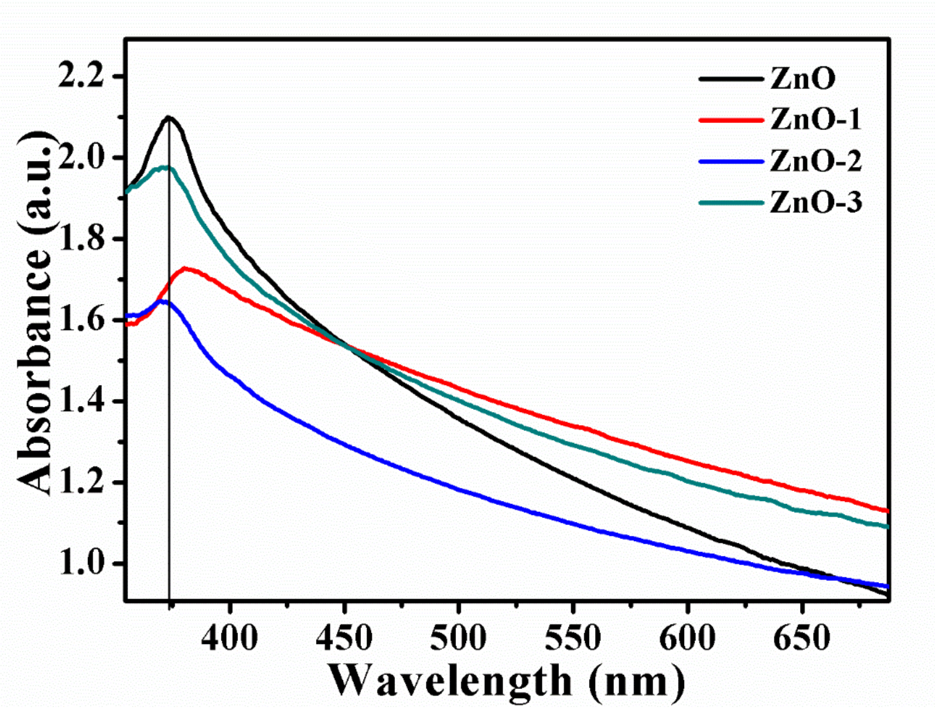


**Figure S3:** UV-Visible Spectra of the ZnO/NiO samples exhibiting absorption peak at 373 nm and a bandgap of 3.32 eV.
